# The Treatment of Enuresis.—II

**Published:** 1909-07-31

**Authors:** 


### Diseases of Children.

### THE TREATMENT OF ENURESIS.—II.

In the previous article on this subject it was stated that the urine is sometimes hyperacid. These cases are generally those of strong, full-blooded, well-fed children, over five years of age, and of the acquired variety. More commonly the urine is alkaline or neutral, of low specific gravity, excessive in quantity, and containing triple phosphates or oxalates, a few pus cells, and a trace of albumin (Lewis). On these grounds certain dietetic measures are recommended. When the urine is highly acid give plenty of water, citrate of potash, and farinaceous food, cutting down the meat supply. As an instance of the uncertainty of treatment, it may be mentioned that Still recommends that the carbohydrates should be cut down, if there is excess of uric acid. If the urine is alkaline it is generally advisable to reduce the amount of carbohydrate food and increase the nitrogenous elements in the diet. It may be taken that the dietetic treatment consists in giving a diet which conforms to the physiological needs of the child and its digestive capacity, paying attention to individual peculiarities. Alcohol, tea, coffee, beef tea, highly seasoned, irritant, and diuretic foods should be omitted. An excess of sugar and sweets is certainly injurious, probably by setting up carbohydrate dyspepsia or intestinal irritation, and upsetting the balance of economy in the supply of other foods by impairing the appetite. Indigestible foods, such as new or imperfectly cooked potatoes, and raw fruits, should be condemned. An ordinary mixed diet is the great desideratum, with attention to digestion and constipation, the promotion of elimination, and measures directed to improving the general health. It is often advantageous to teach the child to defæcate before going to bed, rather than at the usual hour in the morning.

Cold bathing and douching are of value as stimulants of the nervous system and through the psychical effect. Prendergast tried cold douching on 80 boys in an orphan asylum and cured 75, no other treatment being used. A nervous lad was cured by one shock. The treatment is only suitable after the fourth year of age. Strip the child and stand him in a tub or bath. Fill a watering-can or large jug with cold water and pour it over the shoulders and down the back, dry quickly with a coarse towel. Douche every night at bedtime. The ordinary cold bath should be given in the morning to tone up and invigorate the muscular and nervous systems, if the child is strong enough.

Among general measures may be mentioned limitation of the amount of fluid during the latter half of the day, none being allowed after 4 P.M., unless the urine is highly concentrated; a well ventilated bedroom; bedclothes light and not too warm; the lower end of the bed raised; a cotton reel fixed with a tape so that it rests on the spine and prevents the child sleeping on the back; and waking the child to pass water an hour to an hour and a half after going to sleep.

Numerous drugs have been recommended, and their value is most difficult to estimate. On the whole belladonna and atropine have stood the test of time best. It is essential to diagnose as accurately as possible the causation. If there is increased irritability of the lumbar centres, bromides may prove beneficial. Strychnia and its preparations are indicated, if there is lowered cerebral control or atony of the musculature of the bladder. Whether belladonna or atropine is used, it must be pushed up to the limit of tolerance, or failure results, and it must be remembered that children are almost always, but not invariably, extremely tolerant of this drug. It induces flushing of the face, dryness of the tongue and fauces, dilatation of the pupils, and dimness of vision, occasionally an erythematous rash, and even delirium. The last two symptoms show that the dose must be reduced. Give the medicine after tea and before going to bed, in two doses. Begin with five minims, and increase each dose by the addition of two and a half minims every fifth day until 20 minims are given. Keep up the maximum dose for two weeks, and reduce it in the same way as it was increased. Tincture of lycopodium may be added in equal doses, or the belladonna may be combined with nux vomica or bromide. Belladonna is said to contain hyoscyamine and to be safer than atropine. The latter drug is often preferred because of the greater accuracy in dosage. One grain of the sulphate in an ounce of water forms a mixture of which one minim may be regarded as equivalent to one five-hundredth of a grain of alkaloid. Two doses are given at 4 and 7 P.M., and one of the doses is increased every day until the child takes at each dose an amount equal to one minim for each year of its age. It is reduced after the enuresis has ceased for two weeks. Donald Macalister some years ago recommended a mixture of liq. atropin. sulphat. ♏90, liq. strychnin. hydrochlor. ♏45, syr. aurantii ad unciam unam. Five drops are given at 9 P.M., and the dose is increased by five drops every fourth night until it reaches thirty drops, after which it is reduced by ten drops every fourth night. The dose may even be increased to sixty drops. One minim of the liquor atropinæ is equivalent to twenty of the tincture of belladonna, so these large doses should be used with great caution. The tincture may be ordered in doses to half a drachm nightly, and even to a drachm three times a day, especially if combined with citrate of potash and cold douching. The course of treatment lasts for three months, the maximum dose being given for one month and reduced gradually. Antipyrin in large doses, at 7 and 9 P.M. for a week, and repeated after a week's interval, is occasionally curative. The tincture and fluid extract of rhus aromaticus, ♏10-30, are also useful for vesical atony, but not nearly so much so as the arseniate of strychnia. Salol or urotropin is given for bacteriuria and cystitis. Alkalies and nux vomica, ergot and nux vomica, the oxide or valerianate of zinc, and the triple valerianates have also proved beneficial. Finally the habit may be broken in hopeless cases by a course of Weir-Mitchell treatment; by dilatation of the bladder under anæsthesia; by the passage of full-size metallic bougies; or by electrical applications.

